# Rapid Decoding of Hand Gestures in Electrocorticography Using Recurrent Neural Networks

**DOI:** 10.3389/fnins.2018.00555

**Published:** 2018-08-27

**Authors:** Gang Pan, Jia-Jun Li, Yu Qi, Hang Yu, Jun-Ming Zhu, Xiao-Xiang Zheng, Yue-Ming Wang, Shao-Min Zhang

**Affiliations:** ^1^State Key Lab of CAD&CG, Zhejiang University, Hangzhou, China; ^2^College of Computer Science and Technology, Zhejiang University, Hangzhou, China; ^3^Department of Neurosurgery, The Second Affiliated Hospital of Zhejiang University, Hangzhou, China; ^4^Qiushi Academy for Advanced Studies, Zhejiang University, Hangzhou, China

**Keywords:** brain-computer interface, electrocorticography, neural prosthetic control, neural decoding, motor rehabilitation

## Abstract

Brain-computer interface (BCI) is a direct communication pathway between brain and external devices, and BCI-based prosthetic devices are promising to provide new rehabilitation options for people with motor disabilities. Electrocorticography (ECoG) signals contain rich information correlated with motor activities, and have great potential in hand gesture decoding. However, most existing decoders use long time windows, thus ignore the temporal dynamics within the period. In this study, we propose to use recurrent neural networks (RNNs) to exploit the temporal information in ECoG signals for robust hand gesture decoding. With RNN's high nonlinearity modeling ability, our method can effectively capture the temporal information in ECoG time series for robust gesture recognition. In the experiments, we decode three hand gestures using ECoG signals of two participants, and achieve an accuracy of 90%. Specially, we investigate the possibility of recognizing the gestures in a time interval as short as possible after motion onsets. Our method rapidly recognizes gestures within 0.5 s after motion onsets with an accuracy of about 80%. Experimental results also indicate that the temporal dynamics is especially informative for effective and rapid decoding of hand gestures.

## 1. Introduction

Brain-computer interface (BCI) is a direct communication pathway between brain and external devices (Wolpaw et al., 2002). BCI systems do not depend on peripheral nerves and muscles, and thus have great potential to provide new rehabilitation options to patients with motor disabilities (Daly and Wolpaw, 2008), toward the big vision of cyborg intelligence (Wu et al., 2013, 2016; Yu et al., 2016). Electrocorticography (ECoG)-based BCI systems, i.e., the semi-invasive BCIs, have better long-term stability than invasive BCIs (Pilcher and Rusyniak, 1993), although neural spikes (Qian et al., 2018; Xing et al., 2018) have high temporal resolution, and contains richer information than traditional non-invasive BCIs, such as EEG (Blankertz et al., 2004; Sun et al., 2016), thus have been considered as an ideal option for applications such as neural prosthesis control (Leuthardt et al., 2004; Schalk et al., 2008).

A key problem in BCI-based neural prosthesis control is decoding movement intentions from brain signals. Hand gestures convey rich information in communication, and hand gesture decoding has attracted a lot of attention recently. Most existing hand gesture decoding approaches fall into two categories: finger movement regression and hand gesture classification. Some typical studies on hand gesture decoding are summarized in Table 1. Finger movement regression approaches aim to predict the flexion trajectories of individual fingers (Kubánek et al., 2009; Miller et al., 2012, 2014; Xie et al., 2018). But the flexion trajectories of individual fingers in those studies were generated by the movement of single finger. Very few studies tried to decode flexion trajectories of fingers when multiple fingers move simultaneously (Acharya et al., 2010). According to several finger movement decoding studies, the sites of useful signals in ECoG locate separately in space for different fingers (Miller et al., 2012, 2014). When multiple fingers move simultaneously, although the mixed signals of multiple finger movements could be recorded by ECoG electrodes, the temporal overlapping and spatially sparse sampling makes it difficult.

**Table 1:** Hand gesture decoding methods using ECoG signals.

**Authors**	**Problem**	**Gestures**	**Method**	**Window^a^**	**Subject Num**	**Result**
Kubánek et al., 2009	Regression	5 - single finger movement	Linear multivariate decoder	[0, 1.2 s]	5	Average CC^c^–0.63
Acharya et al., 2010	Regression	2 - slow grasping motions of the hand	Generalized linear model	[0, 2 s]	4	Average CC^c^–0.48
Miller et al., 2012	Regression	5 - single finger movement	Generalized linear model	[–1 s, 2 s]	14	Relationship of cortical population activity
Xie et al., 2018	Regression	5 - single finger movement	CNN+LSTM	[0, 1 s]	3	Average CC^c^–0.49
Yanagisawa et al., 2011	Classification	3 - rock, scissors, paper	Linear classifiers	[–2 s, 2 s]	1	79.6%
Chestek et al., 2013	Classification	5 - four finger movements, rest	Naive Bayes	[–0.5 s, +1.5 s]	3	P1-68%, P2-84%, P3-81%
Bleichner et al., 2016	Classification	4 - D, F, V, Y^b^	Template matching	[–1 s, 2 s]	2	P1-97%, P2-74%
Branco et al., 2017	Classification	4 - D, F, V, Y^b^	Template matching	[–1 s, 2.6 s]	5	85%
Li et al., 2017	Classification	3 - rock, scissors, paper	SVM	[0, 1.2 s]	3	P1-85.7%, P2-84.5%, P3-69.7%

a *The movement onset time is regarded as time 0*.

b *American Sign Language finger spelling alphabet D, F, V and Y, respectively*.

c *CC is the abbreviation of correlation coefficients*.

Instead of predicting the flexion trajectories of fingers, hand gesture classification directly regards hand posture decoding as a classification problem, which is more straightforward for practical solution of prosthesis control. Yanagisawa et al. (2011) proposed a real-time decoding system to classify three hand gestures with a linear classifier. Chestek et al. (2013) proposed to use naive Bayes decoder to effectively classify five hand postures from the ECoG signals. These approaches addressed the strength of ECoG signals in hand gesture classification, however, they extracted features using statistics over a long time window, and thus ignored the dynamics in time. Since the performing of gesture is a process, temporal information in ECoG signals contains potential information for decoding. To capture the temporal information, Bleichner et al. (2016) and Branco et al. (2017) proposed a temporal template matching method to decode four gestures from ECoG signals, and Li et al. (2017) proposed SVM-based short-term window approach to further explore the information in time. With short-term time windows, the temporal patterns of different gestures can be characterized, which provides useful information to improve the accuracy in gesture decoding. However, the sequential relationship among windows was not explicitly modeled for accurate decoding. It is still a problem to further exploit the underlying temporal patterns and structures in ECoG signals to improve gesture decoding.

In this study, we propose an RNN-based decoder to accurately recognize hand gestures in ECoG signals. To capture the underlying temporal information in ECoG signals, we propose to use gated RNN models, i.e., long short-term memory (LSTM) models, to learn the temporal patterns of different gestures. The LSTM model can sequentially update the gates in memory cells to determine which features in the preceding windows should be considered for gesture decoding. To benefit temporal pattern learning, our method selects the most temporally informative features to be input to the LSTM decoder. Specially, we evaluate the features in different channels and frequencies by their decoding performances in temporal patten representation, and select the optimal features using a greedy strategy. Experimental results of two subjects show that our method outperforms other methods with an accuracy of 90% in three gesture recognition. Moreover, we investigate the possibility of recognizing the gestures in a time interval as short as possible after motion onsets. The motion intents can be rapidly recognized within 0.5 s after motion onset. Our method achieves high motion recognition performance with quick response, and is promising for online BCI control of prosthetic and robotic devices.

## 2. Methods

**Figure 1:**
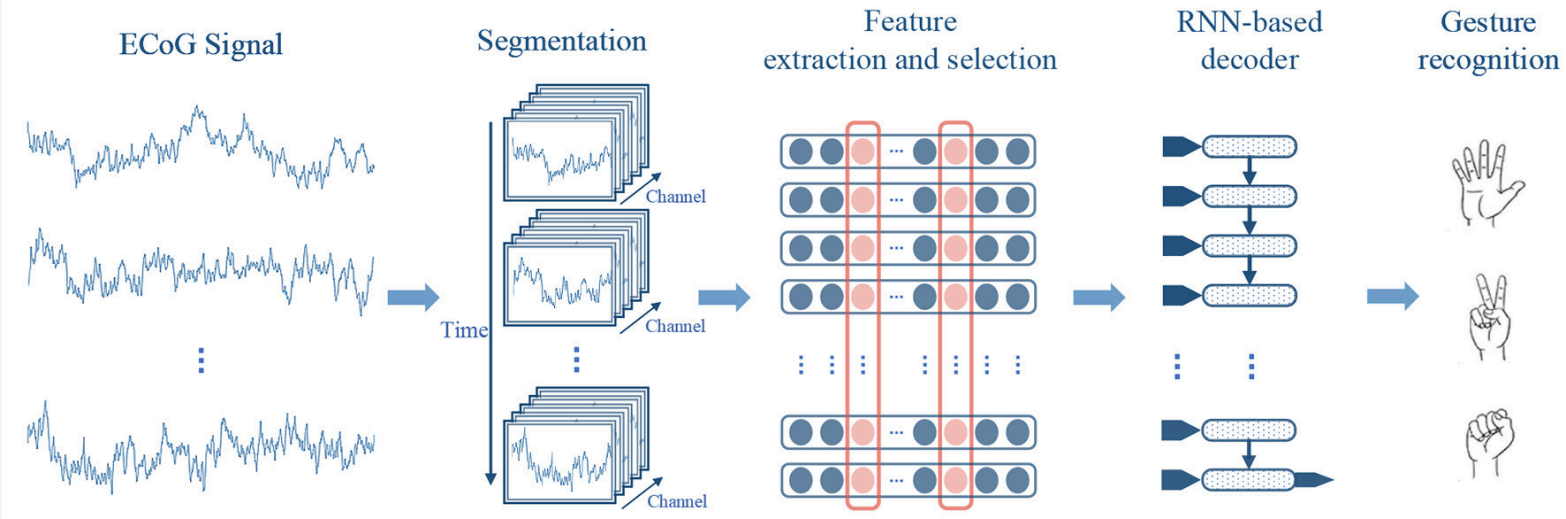
Framework of the proposed method.

The framework of our method is shown in Figure 1. In our approach, the ECoG signals are firstly divided into sequential short-time segments, and power spectrum features are extracted from each segment. Then we select the most informative signal channels along with the frequency bands using a greedy strategy, to compose compact features for decoding. Finally, the features of the segments are sequentially put into a RNN-based decoder for gesture recognition.

### 2.1. Experimental paradigm and data collection

#### 2.1.1. Subjects

**Figure 2:**
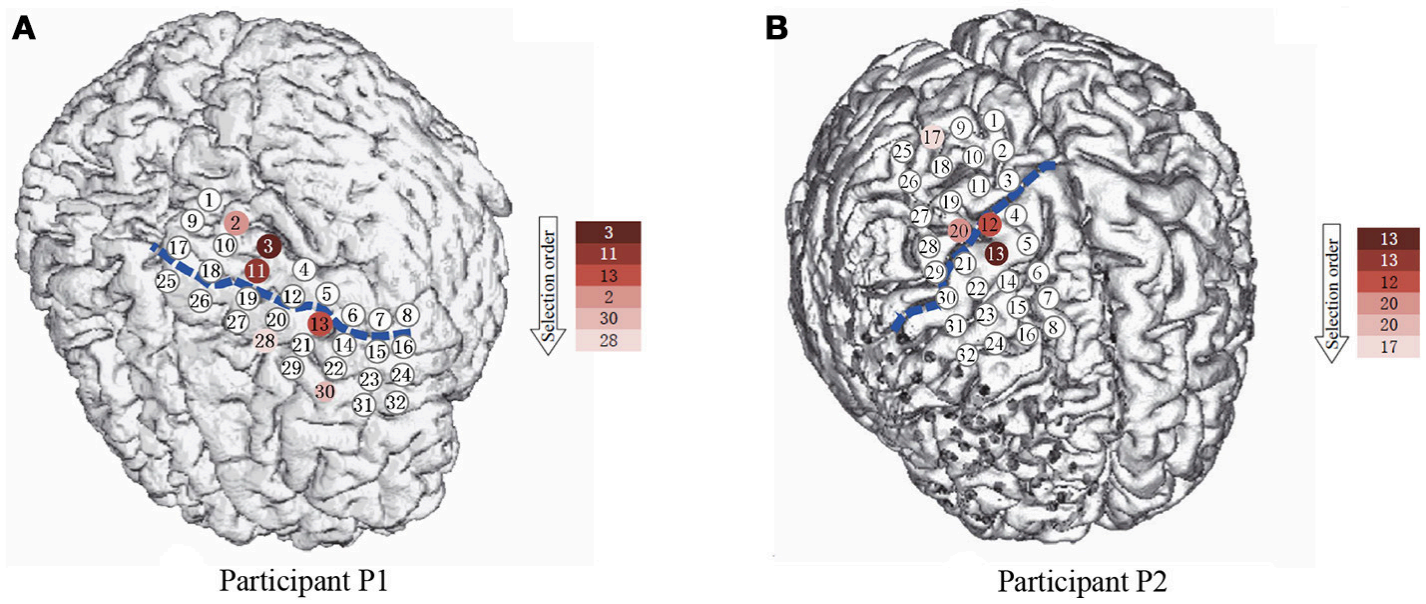
The spatial position of the subdural ECoG electrodes. **(A,B)** are the electrode placement for P1 and P2, respectively. The circles are the position of each electrode, the numbers in the circles present the channels. The blue dash lines mark the central sulcus. The color of the electrodes denote the selection priority in greedy feature selection.

The participants in this study were patients with intractable epilepsy, who had implanted temporary intracranial electrode arrays for surgical purpose. The configuration and location of the electrodes were determined by clinical requirements. The clinical electrodes were platinum electrodes with a diameter of 4 mm (2.3 mm exposed) spacing at 10 mm and generally implanted only for a period ranging from several days up to 2 weeks. Table 2 and Figure 2 presents the information and implantation details of each participant. During the task, the participants temporarily stopped taking the epilepsy medicine under the supervision of doctors. All participants went through the clinical examination routine of the motor, sensory, language function, and so on through cortical stimulation mapping (CSM), which helped to further and functionally localize the electrodes. In addition, combined with preoperative MRI examination, a computed tomography (CT) scans were used to further confirm the location of the electrodes after the implantation surgery, and none of the hand motor areas were in seizure onset zones for both participants. All procedures were followed from the guide and approved by the Second Affiliated Hospital of Zhejiang University, China. Participants gave written informed consent after detailed explanation of the potential risks of the research experiment.

**Table 2:** Information of the participants and electrode locations.

**Participants**	**Gender**	**Age**	**Handedness (task hand)**	Implanted grids^*^	**Seizure focus**
P1	Female	28	Right (right)	LH: temporal, parietal, occipital lobe	Temporal lobe
P2	Male	22	Right (left)	RH: frontal medial, dorsal surface, parietal lobe	Frontal lobe

* *LH, Left hemisphere; RH, Right hemisphere*.

#### 2.1.2. Experimental paradigms

**Figure 3:**
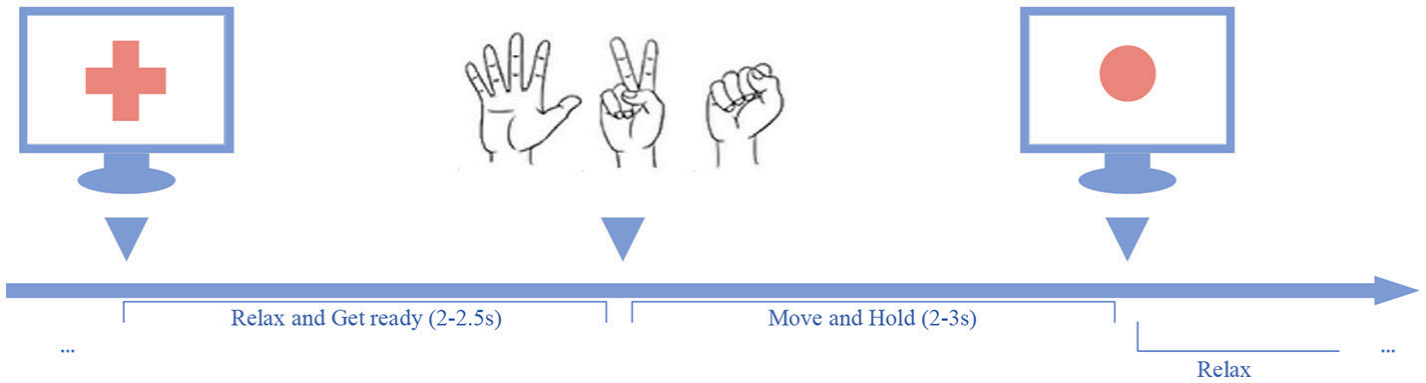
Behavior task paradigm. A trial was initiated by a red cross displayed on the center of the screen along with a verbal cue of “ready”. After a short delay, the red cross disappeared and a gesture cue appeared on the screen, and the participant should perform the given gesture and hold it on, until the red dot appeared.

In the experiment, the participants were asked to perform three kinds of hand gestures (“scissors,” “rock,” and “paper”) guided by the cues presented on the screen. As shown in Figure 3, a trial began with a verbal cue of “ready,” and meanwhile a cross sign displayed at the center of the screen. The cross sign indicates that the participants should relax the task hands and be prepared. During the relax stage, the participants were asked to relax their task hands and flex the fingers slightly with their palms facing up. The relax stage would last for 2–2.5 s randomly. After the relax stage, the cross sign would be replaced by a picture of a randomly selected gesture, and the task stage began. In the task stage, the participants were asked to perform the given gesture instantly, and hold the gesture until a red circle (stop cue) appeared. The task stage would last for 2–3 s randomly. When the stop cue showed, the participants should release the gesture and relax the task hands. At the end of each trial, a verbal feedback “correct” or “wrong” was given by the experimenter to tell the subjects whether it was an eligible trial or not.

**Figure 4:**
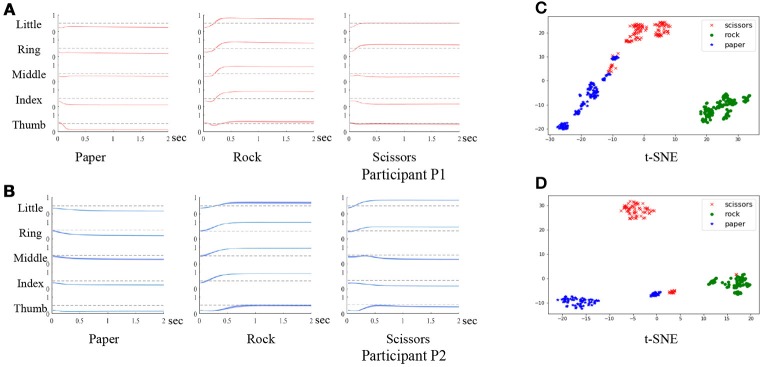
Evaluation of the behavior task. **(A,B)** are the finger trajectories after movement onsets of 3 gestures for participant P1 and P2 (the variance is denoted by the thickness of each line). **(C,D)** are the clustering results of the three gestures using the finger trajectories for participant P1 and P2.

During the experiment, if participants failed to hold the gestures until the stop cue, or forgot to release the gestures, the trial was considered to be invalid. The failed trials were then removed from the dataset. Each session contained three blocks, and each block was composed of 50 trials. For both participants P1 and P2, a total of five sessions were involved in the experiment. The participants would have a short break between the blocks. In practice, the number of trials and the duration of each break depended on the medical condition and the willingness of the participants. Experiments were carried out to evaluate the behavioral compliance of the participants by analyzing the finger trajectories after movement onsets. As shown in Figure 4, the finger movement trajectories are consistent within the same gestures with small variance (denoted by the thickness of the line). We further analyze the trajectories by clustering after t-distributed stochastic neighbor embedding (t-SNE). As shown in Figure 4, the gestures are discriminative for both participants. The results verify the compliance of the behavior task.

#### 2.1.3. Data acquisition

The ECoG signals were collected at the Second Affiliated Hospital of Zhejiang University. The NeuroPort system (128 channels, Blackrock Microsystems, Salt Lake City, UT) was used to record clinical ECoG signals from subdural electrode grids. The recorded signals were stored continuously during the whole task at the sampling rate of 2 kHz and low-pass filtered with a cutoff frequency of 500 Hz. The hand movement data were collected by a 5DT data glove with 14 sensors (5DT Inc., USA) and each sensor simultaneously recorded the finger flexion values. Since we need to mark the onset time of each movement, we defined the onset of a movement as the moment when five first derivative of the flexion values consecutively exceeded a specific threshold. In order to synchronize the neural signals and the motor data, we marked the timestamps of each cue in the ECoG signal recordings using the event channel of the NeuroPort system.

### 2.2. Segmentation and feature extraction

After data acquisition, both ECoG signals and movement signals are continuous. According to the event timestamps recorded synchronously with the signals, the valid trials could be located and preserved for gesture decoding. Each trial contains three timestamps of events: gesture cue start, hand motion onset (indicated by the glove signals), and gesture cue stop.

For each trial, the ECoG signals between “hand motion onset” and “gesture cue stop” is adopted for gesture decoding. The raw ECoG signals are firstly processed by a common average reference spatial filter for noise removal. For each channel, we calculate the average value of the data of the whole session, then the average is subtracted from the raw signals. After filtering, a sliding window is adopted to divide the signals in trials into small temporal segments. In accordance with previous work (Li et al., 2017), we use a window with length of 300 ms and stride of 100 ms. With the temporal segments, the dynamics during the movement stage could be preserved for further decoding.

Then, the power spectral density (PSD) is estimated for each temporal segments. The PSD is calculated using the Welch's algorithm (Welch, 1967). Since the range of the power in different frequency bands could be different, normalization is required. In our method, we adopt the ECoG signals in the relax stage to provide the baseline for normalization. For each channel, we firstly calculated average PSD of all the data segments obtained in relax period:

(1) $$\begin{array}{l} {\overset{-}{R}}_{c,f}=\frac{1}{N}\overset{N_{relax}}{\underset{i=1}{\sum }}R_{c,f}\left(i\right), \end{array}$$

where *R*_*c, f*_(*i*) is the PSD of channel *c* and frequency *f* in the relax segment *i*, and *N*_*relax*_ is the total number of segments in the relax stages. Then PSD of the task signals could be normalized by dividing the respective PSD value in $\overset{-}{R}$:

(2) $$\begin{array}{l} S_{c,f}\left(i\right)=\frac{S_{c,f}\left(i\right)}{{\overset{-}{R}}_{c,f}},\;i=0,1,2,\ldots ,N_{task}, \end{array}$$

where *S*_*c, f*_(*i*) is the PSD of channel *c* frequency *f* in the task segment *i*.

After normalization, we aggregate the PSD values in frequency bands. According to previous studies (Li et al., 2017), a total of five frequency bands are used: a low-frequency band (4–12 Hz), beta frequency band (12–40 Hz), low gamma frequency band (40–70 Hz), high gamma frequency band (70–135 Hz) and a high frequency band (135–200 Hz). For each frequency band, we calculated the average PSD for each channel:

(3) $$\begin{array}{l} {\overset{-}{S}}_{c,t,F}=\frac{1}{F}\overset{N}{\underset{f=1}{\sum }}S_{c,t}\left(f\right), \end{array}$$

where ${\overset{-}{S}}_{c,t,F}$ is the average PSD of *t*th in band *F* for channel *c*, and *F* is the total number of frequencies in each band.

At last, we put extracted features from small temporal segments in a trail into a matrix with *t* rows and *n* columns as a input sequence, where *t* is the number of windows and *n* is the number of features. Each input sequence contains *t* time steps, and *n* features at each time step. This operation let us able to put features into RNN-based model in a recurrent way, which better characterized temporal information by preserving the sequential information in short-term windows. With the temporal segments, the dynamics during the movement stage could be preserved for further decoding.

### 2.3. Gesture recognition

Since the electrode placement was determined by surgery requirements, most channels are unrelated to hand motor activities. The unrelated signals can bring noise in gesture decoding and cause unnecessary computational costs. Therefore, effective feature selection strategy is applied to choose the most informative features for effective and efficient gesture recognition.

#### 2.3.1. Feature selection

In feature selection, we adopt a greedy strategy-based method to select the most informative channels along with the frequency bands. The greedy strategy performs in an iterative manner. Firstly, we choose the feature with the highest decoding performance using an SVM classifier, and put it into the selected set. Then, at each step, we iteratively choose one candidate feature that improves accuracy the most when combined with the selected features, to be added to the selected set. Since the candidate feature is evaluated together with the selected features, redundant features are not likely to be selected. The iteration stops when the request feature number is reached or there is no improvement of decoding performance after adding the newly selected feature. The greedy feature selection strategy is presented in Algorithm 1.

**Algorithm 1:** Greedy Feature Selection

**Input:** Input Feature Matrix *F* containing *N* samples of feature vector ${\left\{f_{i}\right\}}_{i=1}^{N}$
**Output:** Selected Feature List *l*
1: Step 0: Initialization
2: Put *i*th feature with the best accuracy into list *l*
3: *l* ← argmax_*i*_ *P*(*f*_*i*_)
4: Initialize the best accuracy *B* ← 0
5: Initialize local best accuracy *LB* ← *P*(*f*_*l*_)
6: Delete *f*_*i*_
7:
8: Step 1: Greedy Feature Selection
9: **while** *LB* > *B* **do**
10: *B* ← *LB*
11: *l* ← argmax_*i*_ *P*(< *f*_*i*_, *f*_*l*_ >)
12: *LB* ← *P*(*f*_*l*_)
13: Delete *f*_*i*_
14: **return** *l*

#### 2.3.2. Recurrent neural network-based gesture recognition

After feature selection, the feature representation of a task trial can be denoted as {x_1_, x_2_, …, x_*t*_}, where *x*_*i*_ is the feature vector at the *i*th temporal segments. The feature representation takes rich information in both spectrum and temporal dynamic for gesture recognition. Since most classifiers require inputs in the form of vectors, the decoders based on such classifiers need to concatenate the temporal features into a vector. This procedure loses the temporal structure of data, thus leads to inaccurate decoding.

**Figure 5:**
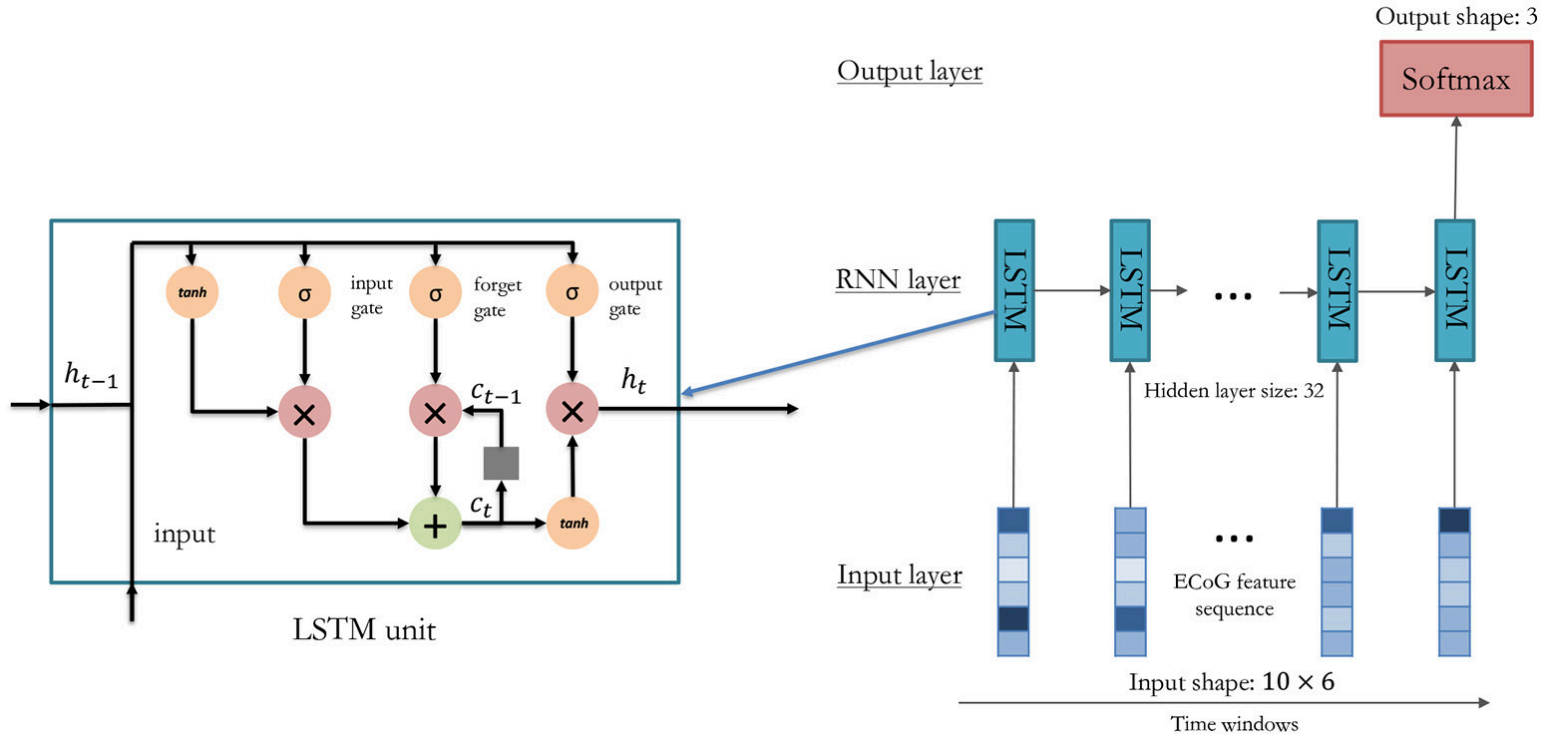
Architecture of RNN model for gesture classification from ECoG.

The RNN-based method overcomes this problem by inputting data in a recurrent way. As shown in Figure 5, the feature vectors are sequentially put into the model, and the temporal information could be well preserved by the temporal connections. In our method, the LSTM model is adopted (Hochreiter and Schmidhuber, 1997):

(4) $$\begin{array}{l} \begin{array}{c} i\left(t\right)=\sigma \left(W_{i}x\left(t\right)+U_{i}h\left(t-1\right)+b_{i}\right),\\ f\left(t\right)=\sigma \left(W_{f}x\left(t\right)+U_{f}h\left(t-1\right)+b_{f}\right),\\ o\left(t\right)=\sigma \left(W_{o}x\left(t\right)+U_{o}h\left(t-1\right)+b_{o}\right),\\ c\left(t\right)=i\left(t\right)tanh\left(W_{c}x\left(t\right)+U_{c}h\left(t-1\right)+b_{c}\right)+f\left(t\right)c\left(t-1\right),\\ h\left(t\right)=o\left(t\right)tanh\left(c\left(t\right)\right) \end{array} \end{array}$$

where *x*(*t*) is the feature vector at the t-*th* time window, *o*(*t*) is the recognition result output from the model after the last time window, σ(*x*) is the sigmoid function, *c*(*t*) is the memory cell, *h*(*t*) is the hidden layer units, and *i*(*t*), *f*(*t*), *o*(*t*) are the input gate, forget gate, and output gate respectively. The memory cell can remember useful information through time, and the gates control how many time windows should be used for the current gesture recognition task. Therefore, in the LSTM model, temporal information can be well preserved for accurate gesture decoding.

## 3. Results

In this section, experiments are carried out to evaluate the gesture decoding performance of our method. Firstly, we examine and analyze the decoding performance of the features selected by different kinds of strategies. Secondly, we test the RNN model with different settings to select the optimal parameters for gesture decoding. After that, the RNN-based decoder is compared with four other competitors to demonstrate the advantages of our method. Finally, we investigate the decoding performance in a time interval as short as possible after motion onsets for rapid gesture recognition. The RNN model is implemented with Keras on the top of TensorFlow.

In the experiment, we have rejected the trials with move artifacts or electrode failures by visual inspection. After removing invalid trails, the dataset includes 243 samples for P1, and 394 samples for P2. In our study, there are a total of three classes of gestures of “rock,” “scissors,” and “paper.”

### 3.1. Feature analysis

In this section, we analyze the features extracted from the ECoG signals. Firstly, we evaluate the feature selection strategy and assess its influence on the gesture recognition performance. Then, experiments are designed to find the appropriate number of features to be applied in gesture recognition. After that, the channels and frequency bands selected are presented and analyzed.

The performance of the greedy-based feature selection is evaluated in comparison with other methods. Firstly, we evaluate the gesture recognition performance using all the channels and frequency bands by the SVM classifier to serve as the baseline in the experiment. Then, an optimal-based feature selection strategy, which independently selects the top *N* features with the best decoding performance, is implemented and compared. The settings of competitors in this experiment are as follows:

- **Baseline:** all the channels with all five frequency bands are used for gesture recognition. The features in temporal segments are concatenated to a vector and put into the SVM classifier.
- **Optimal-based feature selection:** a feature selection strategy that evaluates each frequency of each channel independently, and selects the best *N* features for gesture recognition.
- **Greedy-based feature selection:** our method. The strategy is described in the Algorithm 1.

In this experiment, the signals are divided into temporal segments using a 300 ms sliding window with a stride of 100 ms, and a total of 10 temporal segments following the movement onsets are used. The performance is presented in the average accuracy of 3-fold cross-validation. In gesture classification evaluation, we apply 10-fold cross-validation, for each fold in cross-validation, we randomly select 20% of the training dataset as validation dataset to select the hyper-parameters.

**Figure 6:**
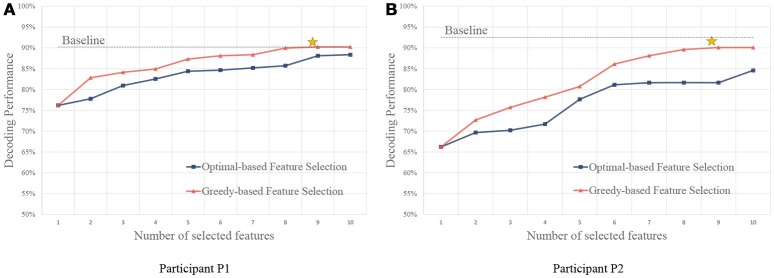
Performance of feature selection strategies using different number of features. **(A,B)** are for participant P1 and P2, respectively. The yellow stars indicate the points that the greedy algorithm stops, and the performance converges after the points.

As shown in Figure 6, we compare the feature selection strategies using the accuracy of the gesture recognition performance. Results show that the baseline method using all the features obtains high performance. With the feature selection strategies, performance close to the baseline can be achieved using only a small set of features. It is because the useless channels could bring noises in classification. Besides, the large amounts of features (both P1 and P2 have 32 signal channels, the total feature number is the product of the number of channel, the number of frequency, and the number of temporal segment) lead to high computational costs.

We also compare the performance using a different number of features. Compared with the optimal-based strategy, the greedy strategy achieves better performance on both of the participants. In the greedy strategy, since the candidate feature is evaluated together with the selected features, redundant features are not likely to be selected, and thus more informative feature sets could be obtained.

**Figure 7:**
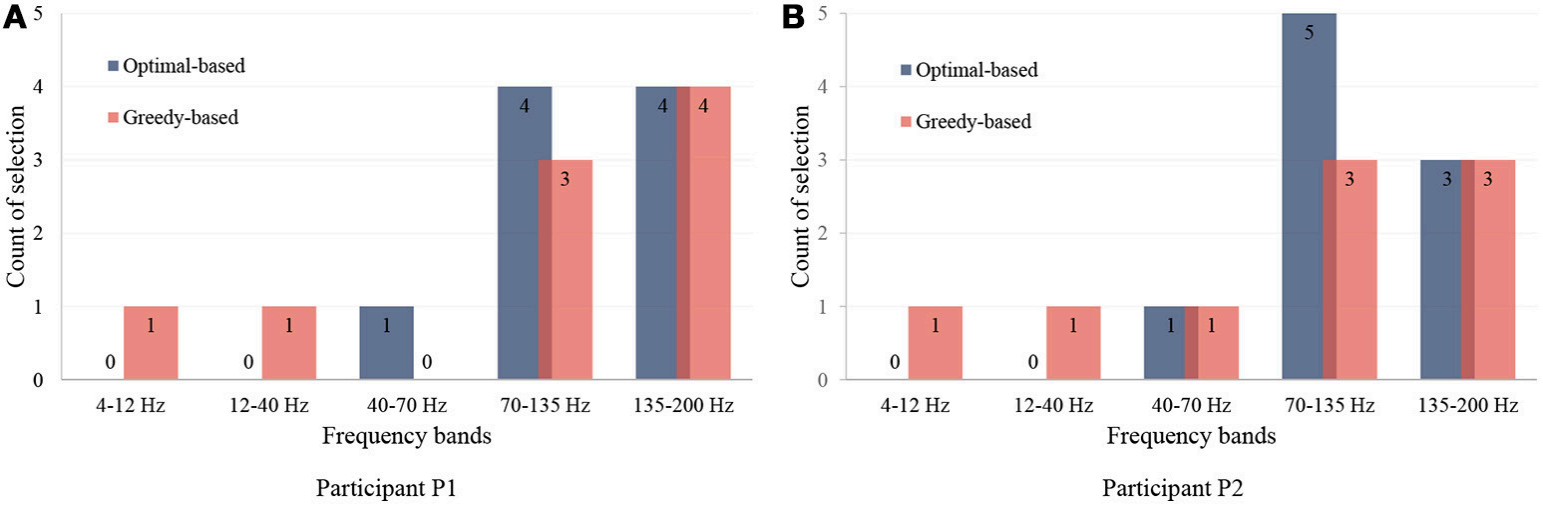
Distribution of selected frequency bands. **(A,B)** are for participant P1 and P2. The first nine features are illustrated and analyzed. The vertical axis presents the number of the selections in each frequency band.

Here, we present the statistical analysis of the channels and the frequency bands selected by our method. The feature distribution of frequency bands is shown in Figure 7, which shows that the most useful bands are 70–135 Hz and 135–200 Hz. The results indicate that high frequency bands in ECoG are highly correlated to hand motions, which is in agreement with previous studies (Bleichner et al., 2016; Branco et al., 2017).

For the number of features, we only used the first six features selected by the greedy algorithm. It is because, although using more features can still lead to improvement of performance as in Figure 6, the later selected electrodes can not bring much improvement. Besides, since the dataset is small, a slight improvement can be brought by overfitting instead of useful information. The channels and their corresponding frequency bands are shown in Table 3. The corresponding electrodes for the features are illustrated in Figure 2. Most of the selected electrodes are close to the central sulcus and within the sensorimotor region, which is in accordance with existing studies (Li et al., 2017).

**Table 3:** The channels and frequency bands selected by the greedy-based strategy.

**Selection order**	**Participant P1**		**Participant P2**	
	**Channel**	**Frequency band (Hz)**	**Channel**	**Frequency band (Hz)**
1	3	70–135	13	70–135
2	11	135–200	13	135–200
3	13	135–200	12	70–135
4	2	135–200	20	12–40
5	30	70–135	20	135–200
6	28	135–200	17	70–135

### 3.2. Performance of gesture recognition

In this section, we evaluated the decoding performance of our method. Firstly, in order to maximize the performance of the classifiers, experiments are carried out on the validation dataset to select the optimal model settings. Secondly, we compare our method with other decoders to demonstrate the effectiveness of temporal information, and the ability of RNN in ECoG time series decoding.

#### 3.2.1. Model selection

Experiments are carried out to select the optimal setting for the LSTM RNN model. For the LSTM model, one important setting is how many hidden units are used. Models with a small set of hidden units may not be useful to encode the information, while models with large sets of hidden units are prone to overfitting.

**Figure 8:**
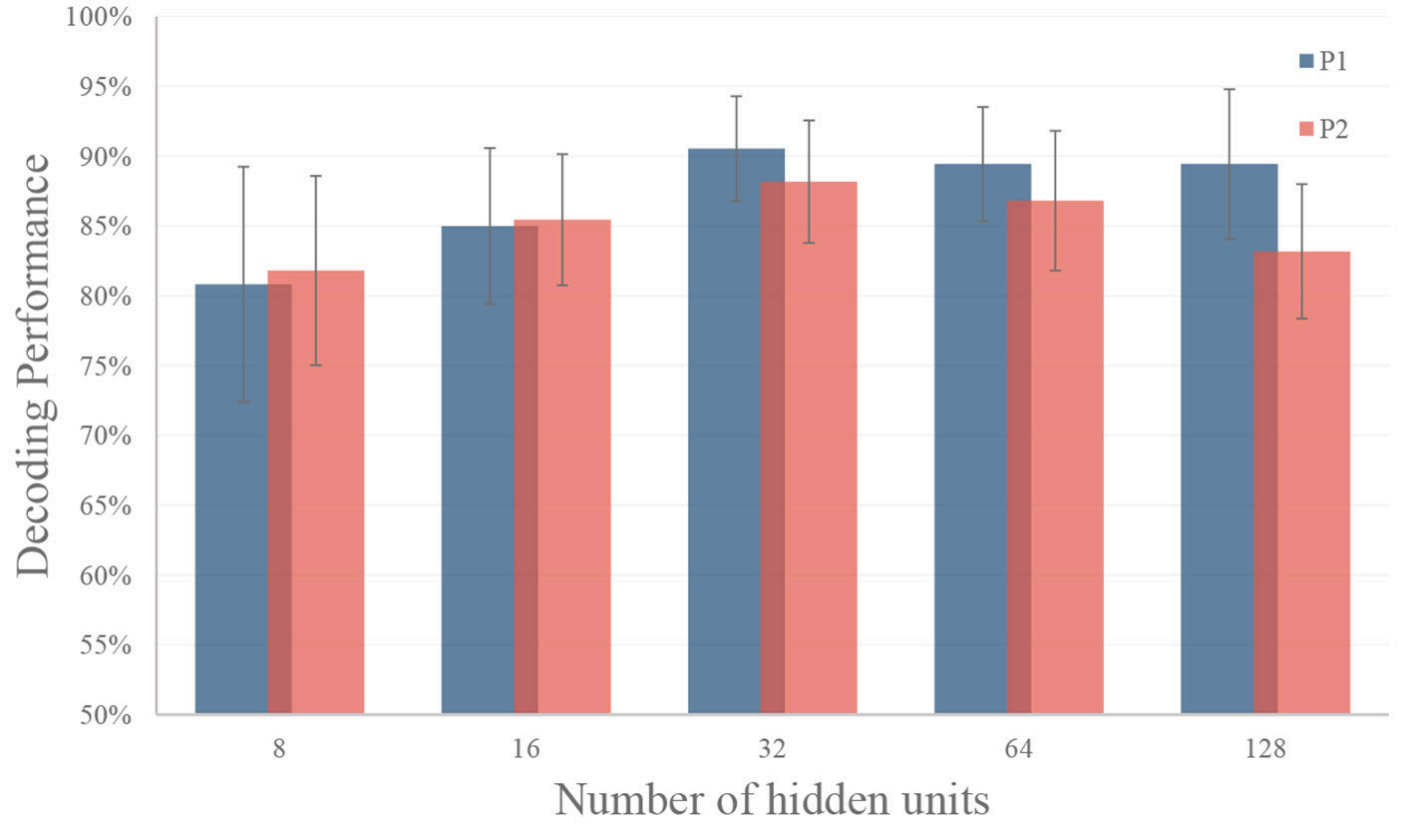
Performance of different number of hidden units in RNN. The LSTM model with 32 hidden units got the best performance (90.56% on for P1 and 88.18% for P2) for both participants on validation dataset. The black dash line represents the standard deviation.

In this experiment, we tune the number of hidden units from 8 to 128 to test the performance of the LSTM model. In this experiment, we use the top six features selected by the greedy strategy, and the settings of temporal segments are the same as in section 3.1. As shown in Figure 8, the LSTM model with 32 hidden units got the best performance (90.56% on for P1 and 88.18% for P2) for both participants on validation dataset. Therefore, we use 32 hidden units for gesture recognition in our decoder. In model training, we use Adam optimization algorithm, the learning rate was set to be 0.001 with a decay rate of 0.0005 for each epoch. An early stop was applied by selecting the epoch with the best performance on the validation set.

#### 3.2.2. Comparison with other methods

In this experiment, comparison is carried out between our method and other decoders. We firstly compare our method with decoders using long time windows to evaluate the effectiveness of temporal information. Then our decoder is compared with other classifiers to demonstrate the strength of RNN models in sequential modeling. For the competitors, we carefully select typical segment-based ECoG/EEG classification approaches from the existing studies, including linear and nonlinear methods. For linear method, we choose the widely used logistic regression method as in Subasi and Erçelebi (2005). For nonlinear method, we choose the classical SVM classifier with RBF kernel as in Li et al. (2017) for comparison. We also compare the segment-based approaches with the method using long time windows to show the effectiveness of temporal information. In order to demonstrate the effectiveness of recurrent structure, we compare our method with an MLP-based approach as in Chatterjee and Bandyopadhyay (2016), to evaluate advantage of weight sharing of RNN models. In this experiment, the signals are divided into temporal segments using a 300 ms sliding window with a stride of 100 ms. A total of 10 temporal segments are used. Thus, each input sequence contains 10 time steps, and 6 features at each time step for our RNN model.

In this experiment, we evaluate our method in comparison with other methods using a permutation test. In each permutation trial, we randomly select 10% of the data for test, and run a total of 500 trials. We also examine the significance of the results using paired *t*-test.

The implementation and settings of the competitors in this experiment are as follows:

- **SVM-Global:** a SVM-based decoder using features calculated over long time windows. For fair comparison, the length of the ECoG signal used is the same as the following competitors. RBF kernel is used in the SVM model, and the parameters of C and gamma is selected by cross-validation. The parameters C is selected from 0.1,1,10,100 and 1,000, and gamma was selected from 0.01, 0.001, and 0.0001.
- **SVM-Segments:** a SVM-based decoder using features in temporal segments (Li et al., 2017). The segment settings are the same as the RNN method. The features in sequence are reshaped into a single vector to input to the SVM classifier. RBF kernel is used in the SVM model, and the parameters of C and gamma is selected by cross-validation. The parameters C is selected from 0.1,1,10,100, and 1,000, and gamma was selected from 0.01, 0.001 and 0.0001.
- **MLP-Segments:** a multilayer perception based decoder from previous work (Chatterjee and Bandyopadhyay, 2016). The segment settings are the same as the RNN method. The features in sequence are reshaped into a single vector to input to the MLP classifier.
- **LR-Segments:** a logistic regression based decoder from previous work (Subasi and Erçelebi, 2005). The segment settings are the same as the RNN method. The features in sequence are reshaped into a single vector to input to the LR classifier.

The results are shown in Table 4. Overall, the RNN-based decoder obtains the highest accuracies for both participants. For participant P1 the gesture recognition accuracy is 89.34%, and for participant P2 the gesture recognition accuracy is 90.83%. Among the competitors, the SVM-Global gives the worst performance. It is reasonable since it calculates the features using the whole time window and ignores the information in time. The SVM-Segments method improves the accuracy by 7.48 and 8.72% for P1 and P2 respectively, by using the temporal segments. The results demonstrate the importance of considering the temporal information in ECoG decoding. The significance of the results are evaluated using paired *t*-test. Results show that our method statistical significantly outperforms other approaches under significance of 0.01 (see Table 5).

**Table 4:** Gesture recognition comparison of different decoders.

**Decoder**	**P1**	**P2**
SVM-Global	79.03% ± 6.50	78.94% ± 7.60
SVM-Segments	86.51% ± 5.23	87.66% ± 6.19
MLP-Segments	84.35% ± 5.76	87.11% ± 7.13
LR-Segments	83.82% ± 5.69	85.76% ± 6.77
RNN (ours)	89.34% ± 4.67	90.83% ± 5.94

**Table 5:** *P*-value of paired *t*-test in comparison with other methods.

**t-test**	**P1**	**P2**
Ours vs. SVM-Global	1.52E-141	6.25E-129
Ours vs. SVM-Segments	3.31E-34	2.37E-25
Ours vs. MLP-Segments	3.08E-66	9.34E-28
Ours vs. LR-Segments	8.58E-86	6.06E-53

### 3.3. Rapid recognition

Quick recognition is an important issue in BCI-based prosthetic control. In this section, we investigate the possibility of recognizing the gestures in a time interval as short as possible after motion onsets. In the experiments, we tune the time interval from 100 ms to 1,200 ms after motion onsets. For each time interval, the ECoG signals are divided using a 300 ms sliding window with a stride of (*t* − *w*)/9 ms, where *t* is the time interval and *w* = 300 ms is the length of the sliding window. If the time interval is < 300 ms, we use a *w* = *t*/2 ms sliding window with a stride of *w*/9 ms. A total of 10 temporal segments are used. We evaluate the performance using a permutation test. In each permutation trial, we randomly select 10% of the data for test, and run a total of 500 trials.

**Figure 9:**
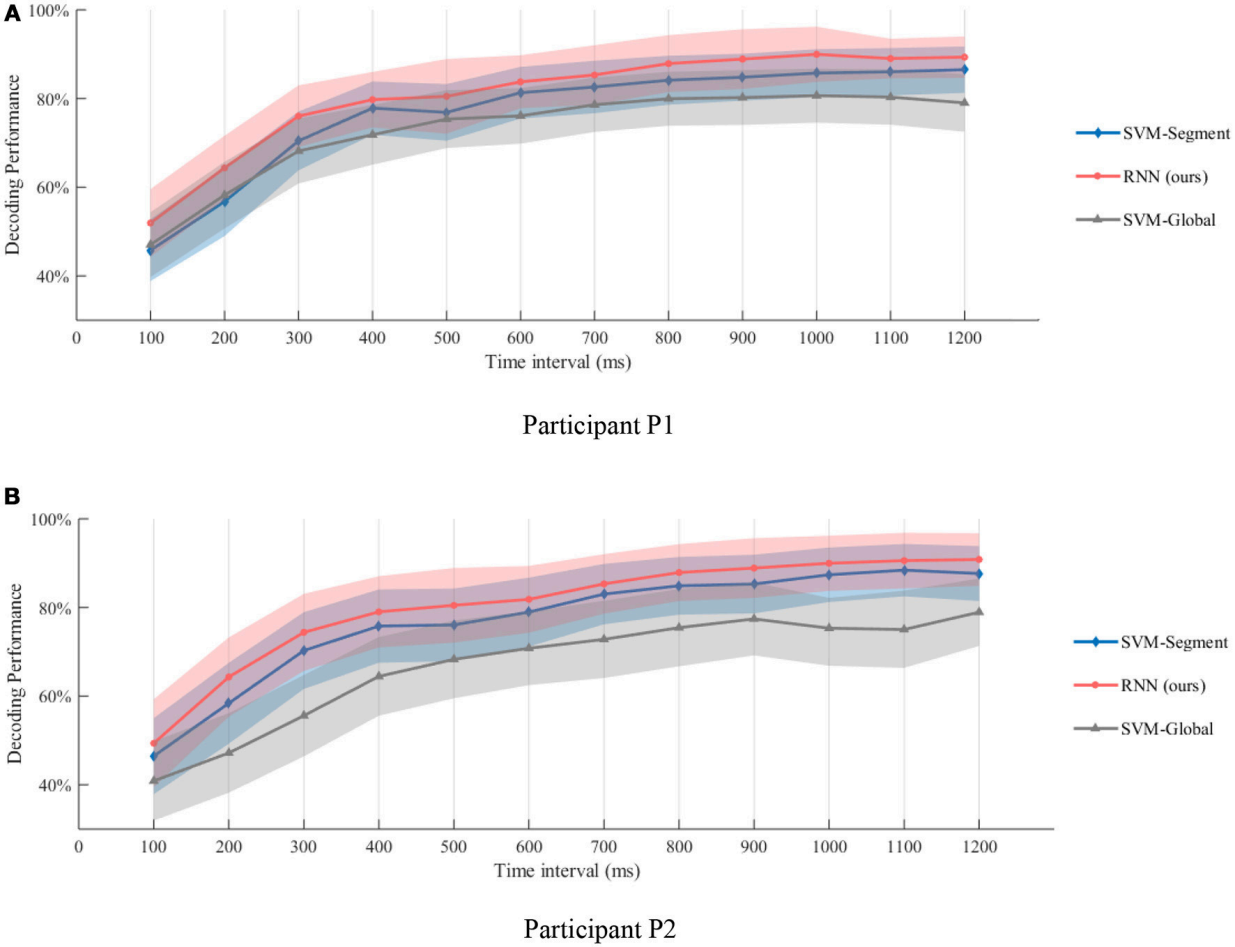
Gesture recognition performance within different time intervals after motion onsets. **(A,B)** are the decoding performance of different methods on Participant P1 and P2, respectively. Recognition accuracies of over 75% could be obtained at the 0.3 s interval for both of the participants. If we use a 0.5 s time interval, the gesture recognition accuracy is over 80%. The results also indicate that, the temporal dynamic is especially informative for quick decoding within short time intervals.

The results are shown in Figure 9. As the time interval become longer, better gesture decoding performance could be obtained. The results of this experiments also demonstrate the possibility of rapid recognition. As shown in Figure 9, recognition accuracies of over 75% could be obtained at the 0.3 s interval for both of the participants. If we use a 0.5 s time interval, the gesture recognition accuracy is over 80%. The results also indicate that, the temporal dynamic is especially informative for quick decoding within short time intervals. The significance of the results is evaluated using paired *t*-test, and our method outperforms both SVM-Global and SVM-Segment significantly with *p* < 0.01. The details of the *t*-test results are shown in the Supplementary Table 1.

## 4. Discussions

In this study, we have shown that ECoG signals provide useful information for effective hand gesture classification, and demonstrated the importance and effectiveness of temporal in formation in gesture decoding. Compared with the existing approaches, our method explore further on the temporal information in ECoG signals to achieve more accurate hand gesture decoding. Bleichner and Branco et al. (Bleichner et al., 2016; Branco et al., 2017) proposed to use temporal template matching of local motor potential (LMP) for each channel for gesture decoding. Compared with their approaches, our method considered temporal information in different frequency bands, and modeled patterns and underlying relationships using the RNN decoder. Li et al. (2017) proposed to model temporal information in the ECoG signals using short-term time windows and SVM classifier. In their approach, the features in temporal sequence were reshaped into a vector for classification, which broke the temporal structure of the features. Different from their method, our RNN-based decoder input features in a recurrent way, which better characterized temporal information by preserving the sequential information in short-term windows. Elango et al. (2017) proposed to use RNN-based models to classify individual finger movements. Different from their approach which manually selected the ECoG channels and frequencies from empirical observations, our method selected the optimal channels and frequencies with a greedy strategy to provide the most useful temporal information for gesture decoding. Overall, our method further exploited the temporal information of ECoG signals in both feature selection stage and gesture decoding stage, and recognized three hand gestures with a high accuracy of 90%. Besides, our results provided evidence for the possibility of rapid recognition. As shown in Table 1, most existing methods require long detection delays (from 1.2 to 2.6 s) to achieve high performance, which leads to poor user experience in real-time prosthesis control. In our system, quick response can be achieved within 0.5 s with an accuracy of 80%, which is promising for online applications.

**Figure 10:**
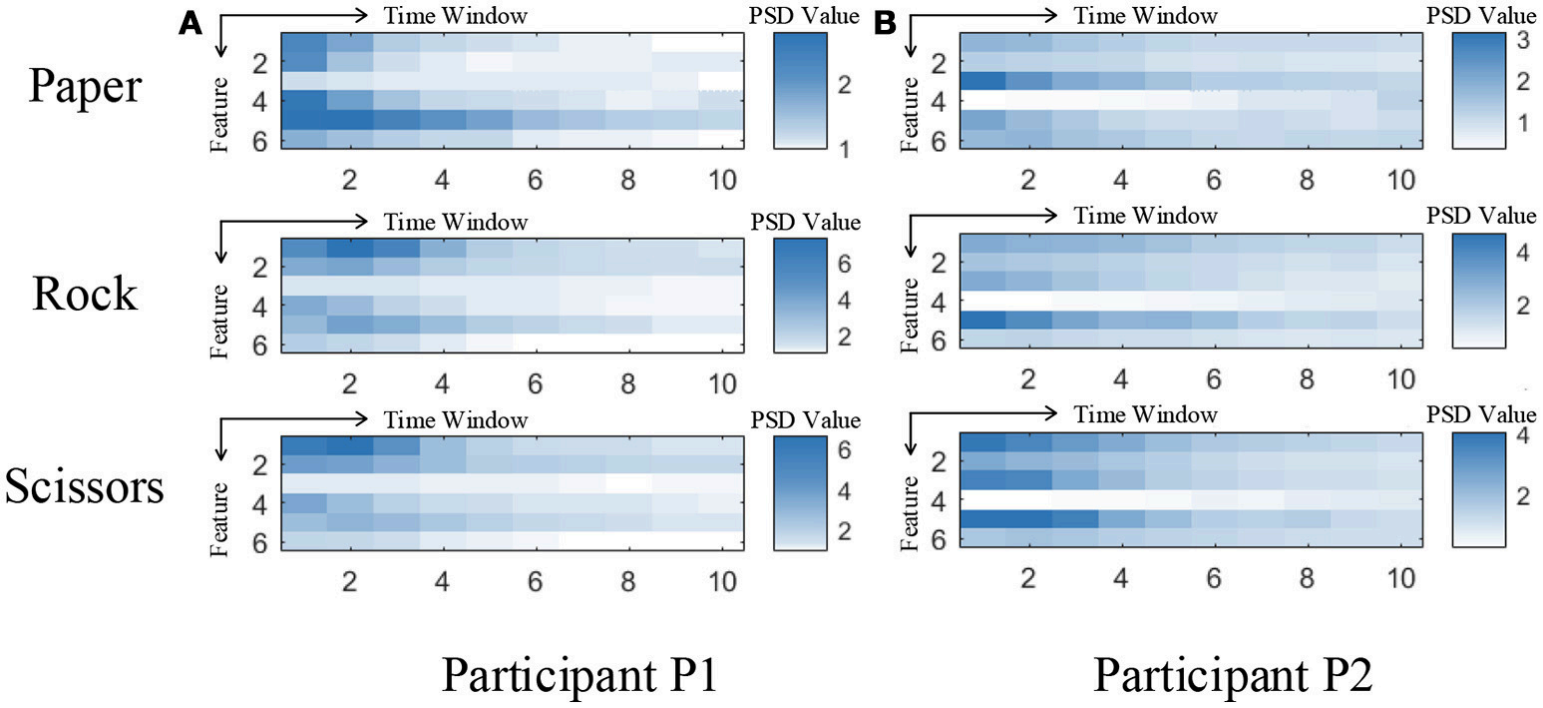
Temporal dynamic of different gestures. **(A,B)** are the feature values of 3 gestures averaged from all the samples for participant P1 and P2, respectively. Each subfigure illustrates the averaged feature values of the six selected features of ten time windows.

**Figure 11:**
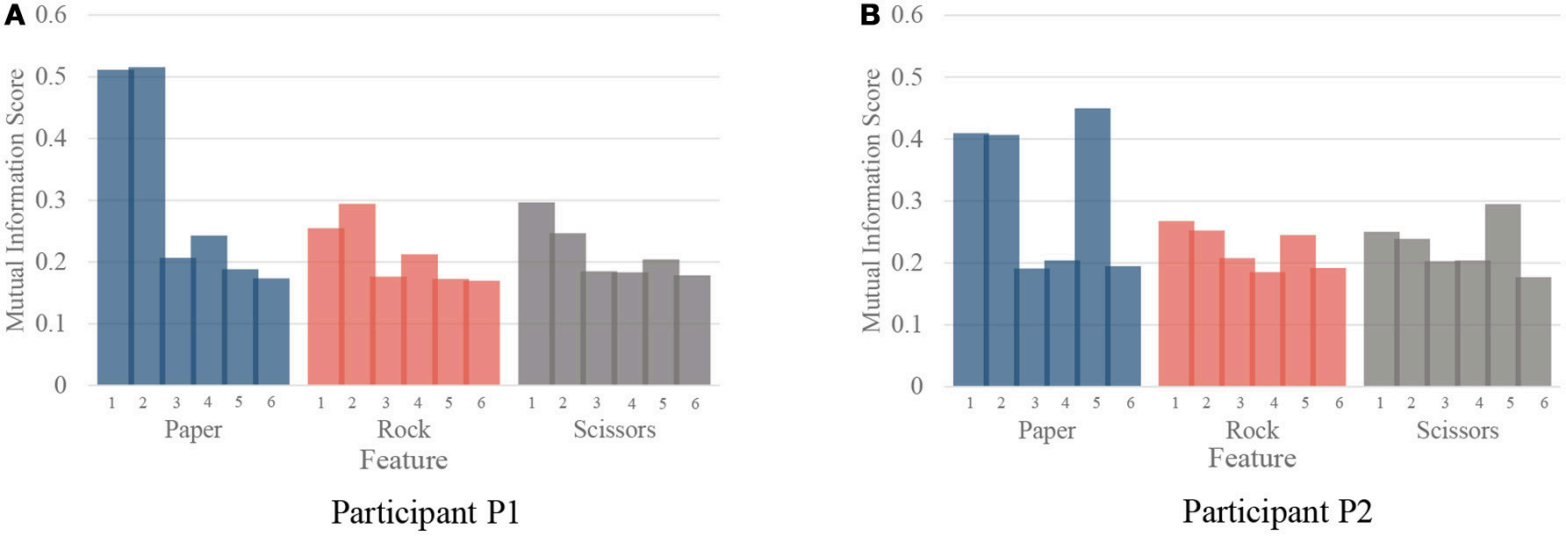
The importance of each feature for different gestures. **(A,B)** are the importance evaluated by mutual information for participant P1 and P2, respectively.

Although our model achieved great results on ECoG signals, the details of temporal information still need a discussion. The temporal dynamic of different gestures is illustrated in Figure 10. The color presents the feature values of six features in different time. The features are ordered by the selection order as in Table 3. The horizontal axis denotes the time windows, where 0 is the movement starting point. As described in section 3, the window length is 300 ms with a stride of 100 ms. It is shown that, the features contain varying patterns in time. Most of the features show higher values in the first several time windows and the values decrease with time. One exception is the fourth feature for P2, which has small values shortly after movement onset. It is reasonable because the feature covers low frequency band (12–40 Hz). The feature might be chosen under overfitting. We also evaluate the importance of each feature for different gestures. In Figure 11, we present the mutual information of each features to the gesture labels. For P1, the most informative features are the 1st and the 2nd (the corresponding electrodes are 3 and 11 respectively), for P2, the most informative features are the 1st, 2nd, and 5th (the corresponding electrodes are 13 and 20 respectively). The most informative electrodes are close to the central sulcus. For P2, although the 5th feature is informative, the selection priority is not high. It might because the feature is correlated to the early selected features. Therefore, it is not preferable in the greedy algorithm. In addition, the results in feature selection show that most of the selected electrodes are distributed along both sides of the postcentral gyrus in two participants, which is in accordance with existing studies (Pistohl et al., 2012; Wang et al., 2012; Chestek et al., 2013). The results suggest that the activation of the postcentral gyrus play an influential role in hand movement. This phenomenon is probably due to the motor control copy or the force-related feedback.

## 5. Conclusion

In this study, we proposed a RNN-based method to exploit the temporal information in ECoG signals for rapid and robust gesture recognition. Compared with the existing approaches using linear methods or SVM classifiers, the RNN model better preserved the structure in feature sequence and was capable of learning from nonlinear relationships. Our system recognized three hand gestures with a high accuracy of 90%, and quick response was achieved within 0.5 s with an accuracy of 80%. The results showed that ECoG signals provide useful information for effective hand gesture classification, and demonstrated the possibility of rapid recognition. The results provided further evidence for the feasibility of robust and practical ECoG-based control of prosthetic devices.

## 6. Ethics statement

This study was carried out in accordance with the recommendations of the institutional review board ethical guidelines of the Second Affiliated Hospital of Zhejiang University with written informed consent from all subjects. All subjects gave written informed consent in accordance with the Declaration of Helsinki. The protocol was approved by the Medical Ethical Committee of the Second Affiliated Hospital of Zhejiang University, China.

## Author contributions

GP and S-MZ conceived and designed the experiment. S-MZ and J-MZ collected and preprocessed the clinical data. J-JL and HY performed the data analysis. GP, YQ, X-XZ, and Y-MW provided advice on the analysis and interpretation of the final results. YQ, GP and J-JL wrote the paper.

### Conflict of interest statement

The authors declare that the research was conducted in the absence of any commercial or financial relationships that could be construed as a potential conflict of interest. The reviewer XT and handling Editor declared their shared affiliation.
