# Supplementary material for: Rapid Decoding of Hand Gestures in Electrocorticography Using Recurrent Neural Networks
Source: Front Neurosci. 2018 Aug 27;12:555. doi: 10.3389/fnins.2018.00555 (PMC6119703; doi:10.3389/fnins.2018.00555)
Supplement: Supplementary file 1 [file Table_1.DOCX]

Supplementary table 1 P-value of paired t-test in rapid recognition.

| Time interval | Ours vs. SVM-Global | | Ours vs. SVM-Segments | |
| --- | --- | --- | --- | --- |
|  | P1 | P2 | P1 | P2 |
| 100 ms | 2.87E-38 | 5.00E-47 | 1.05E-56 | 6.19E-09 |
| 200 ms | 1.43E-66 | 8.02E-133 | 8.42E-78 | 3.56E-36 |
| 300 ms | 4.71E-93 | 8.42E-78 | 2.43E-63 | 6.30E-22 |
| 400 ms | 1.80E-109 | 2.86E-156 | 3.84E-14 | 7.81E-22 |
| 500 ms | 2.35E-25 | 1.95E-114 | 3.88E-14 | 1.77E-38 |
| 600 ms | 1.84E-105 | 7.42E-93 | 6.49E-26 | 1.04E-18 |
| 700 ms | 6.20E-52 | 3.87E-105 | 1.09E-11 | 4.84E-13 |
| 800 ms | 1.45E-67 | 2.28E-115 | 9.46E-22 | 4.44E-24 |
| 900 ms | 1.53E-73 | 1.10E-117 | 1.83E-22 | 3.27E-29 |
| 1000 ms | 9.03E-85 | 1.03E-111 | 7.51E-27 | 1.40E-20 |
| 1100 ms | 8.33E-122 | 9.32E-149 | 1.74E-40 | 3.94E-14 |
| 1200 ms | 1.52E-141 | 6.25E-129 | 3.31E-34 | 2.37E-25 |
